# Risk of end-stage renal disease associated with gout: a nationwide population study

**DOI:** 10.1186/ar3806

**Published:** 2012-04-18

**Authors:** Kuang-Hui Yu, Chang-Fu Kuo, Shue-Fen Luo, Lai-Chu See, I-Jun Chou, Hsiao-Chun Chang, Meng-Jiun Chiou

**Affiliations:** 1 Division of Rheumatology, Allergy and Immunology, Chang Gung Memorial Hospital, 5, Fu-Hsing Street, Kuei Shan, Taoyuan, 333, Taiwan; 2 Department of Internal Medicine, College of Medicine, Chang Gung University, 259 Wen-Hwa 1st Road, Kwei-Shan Tao-Yuan, 333, Taiwan; 3 Division of Academic Rheumatology, School of Clinical Sciences, University of Nottingham, Nottingham, UK (Clinical Sciences Building, Nottingham City Hospital, Hucknall Road, Nottingham, NG5 1PB, UK; 4 Biostatistics Core Laboratory, Molecular Medicine Research Center, Chang Gung University, 259 Wen-Hwa 1st Road, Kwei-Shan Tao-Yuan, 333, Taiwan; 5 Biostatistics Consulting Center, Department of Public Health, Chang Gung University, 259 Wen-Hwa 1st Road, Kwei-Shan Tao-Yuan, 333, Taiwan; 6 Department of Pediatrics, Chang Gung Children's Hospital, 5, Fu-Hsing Street, Kuei Shan, Taoyuan, 333, Taiwan

## Abstract

**Introduction:**

We explored the risk of end-stage renal disease (ESRD) among gout patients in a representative cohort in Taiwan.

**Methods:**

The primary database used was the Taiwan National Health Insurance Research Database. Subjects older than 20 years without ESRD, coronary heart disease, or stroke were included in the study. The case definition of gout in the present study was gout diagnosis and medical treatment for gout. An ESRD case was defined by the presence of chronic renal failure necessitating long-term renal replacement therapy. Multivariate Cox proportional hazards models were used to evaluate the risk of ESRD among gout patients.

**Results:**

The analysis included data of 656,108 patients who were followed up for a mean of 8.0 years. Among them, 19,963 (3.0%) patients had gout. At the end of 2008, 2,377 individuals (gout, *n *= 276; non-gout, *n *= 2,101) had ESRD, and 861 individuals (gout, *n *= 77, 27.9%; non-gout, *n *= 521, 24.8%) died due to ESRD. The rates of incidence of ESRD were 1.73 and 0.41 cases per 1,000 patient-years in the gout and non-gout groups. After adjustment for age, sex, and history of diabetes mellitus and/or hypertension, gout was associated with a hazard ratio (HR) of 1.57 for ESRD (95% confidence interval [CI], 1.38-1.79; *P *< 0.001). In patients with ESRD, the adjusted HR for death in patients with gout was 0.95 (0.74-1.23, *P *= 0.71), which was similar to the HR obtained in patients without gout.

**Conclusions:**

Gout is associated with an increased hazard for development of ESRD.

## Introduction

Chronic kidney disease (CKD) is the progressive loss of renal function over a period of months or years. Continuous deterioration of renal function in patients with CKD eventually leads to the development of end-stage renal disease (ESRD). Patients with ESRD require renal replacement therapy, such as hemodialysis, peritoneal dialysis, or kidney transplantation, for sustenance. ESRD is increasingly becoming a major health burden worldwide [1,2]. The prevalence and incidence of ESRD are alarmingly high in Taiwan in particular [3]. A recent study based on data from the National Dialysis Registry of Taiwan revealed that 48,072 patients required hemodialysis and 4,465 required peritoneal dialysis in 2007; these numbers correspond to a prevalence of 2,288 ESRD cases per population of 1,000,000 [4].

Traditionally, diabetes mellitus (DM) and hypertension have been considered the most important risk factors for CKD [5-7]. Since gout frequently coexists with DM [8] and hypertension [9], it is often overlooked as a genuine risk factor for CKD and subsequent ESRD [10]. Studies in humans have provided limited evidence of the link between hyperuricemia, which is the biochemical hallmark of gout, and deterioration of renal function [11]. A previous study reported the existence of a modest but significant association between the quintiles of uric acid levels and deterioration of renal function [12]. Another study found that the risk for CKD increased 1.69-fold for every 2-mg/dL increase in uric acid levels [13]. Our recent study revealed that hyperuricemia was strongly associated with CKD independently of the presence or absence of the metabolic syndrome [14]. Moreover, subjects with hyperuricemia are more likely to develop ESRD than are those without hyperuricemia [15]. However, the role of gout as an independent risk factor of CKD/ESRD has not yet been explored. By analyzing the data obtained for a representative cohort of the general population in Taiwan, the present study sought to determine whether gout independently increased the hazard of development of ESRD.

## Materials and methods

This study was approved by the institutional review board of the Chang Gung Memorial Hospital in Taiwan (approval number 97-1564C) and was conducted in full compliance with the local and national ethical and regulatory guidelines. The primary data sources of this prospective observational study were the Taiwan National Health Insurance Research Database (NHIRD) and the National Death Registry in Taiwan. This study was supported by a research grant from the Chang Gung Memorial Hospital. The study was designed and the data collected, analyzed, and interpreted by the investigators independently.

### Study population

The Taiwan National Health Insurance (NHI) system was established in 1995 and requires all residents of Taiwan to participate. At the end of 2010, 23.03 million residents of Taiwan were enrolled in this system. The NHI database contains the registration files and original claims data, including comprehensive information on demographics, inpatient and ambulatory care, diagnostic codes, medical expenditure, operations, prescriptions, examinations, and procedures.

The Taiwan NHIRD releases sets of sampling files, called the Longitudinal Health Insurance Database (LHID), for research purposes. LHID 2000 contains the entire original claims data for 1,000,000 beneficiaries randomly sampled from the entire population of NHI beneficiaries in the year 2000. The source population was the entire population covered by NHI (23,753,407 individuals) in the year 2000. The sampling procedure was detailed on the website of National Health Research Institute [16] and is summarized briefly in the following text. Each individual was assigned a serial number ranging from 1 to 23,753,407. By means of a linear congruential random number generation method, 1,000,000 non-duplicated random numbers were generated. Individuals with serial numbers corresponding to these numbers were selected. All registration and claims data for these 1,000,000 individuals, recoded for the period of 1996 to 2008, were collected and distributed as LHID 2000. The subjects included in LHID and those enrolled in the original Taiwan NHIRD do not differ significantly in terms of age, sex, or mean insured amount.

Any insured citizen with a major disease can apply for a catastrophic illness certificate, which entitles the holders to subsidies and waivers of the outpatient and inpatient copayments. The prerequisites for the issuance of this certificate are a diagnosis of catastrophic illness by a physician and a formal review by the Bureau of National Health Insurance. ESRD is also listed as a catastrophic illness.

In this study, we used LHID 2000 as the primary source of data. The eligible criteria included (a) age at least 20 years and (b) the absence of ESRD, coronary heart disease (CAD), and stroke before the year 2000. Subjects were classified by a history of gout before 1 January 2000.

### Case definition of gout

The case definition of gout in the present study consisted of a gout diagnosis (International Classification of Diseases, Ninth Revision [ICD-9] code 274.x) and medical treatment for gout at either an outpatient or emergency department during 1996 to 1999. The definition of medical treatment consisted of the use of colchicine and the use of urate-lowering agents (including benzbromarone, allopurinol, probenecid, and sulfinpyrazone). We also used a secondary case definition (clinical diagnosis with gout in records of two visits to outpatient or emergency departments) for gout in this study. As the results were similar and did not change the overall conclusion, we reported results using the primary case definition. The utility and validity of gout diagnoses in claims data had been assessed. Positive predictive values of gout diagnosis recorded in the database had been reported to range from 61% [17] to 100% [18].

### Definition of outcome

The primary outcome was ESRD. Patients with ESRD require chronic renal replacement therapy, including hemodialysis, peritoneal dialysis, and kidney transplantation, to survive, and this fulfills the requirement for a catastrophic illness certificate in Taiwan. Thus, we identified patients with ESRD by linking the LHID to the registry for catastrophic illnesses.

### Definition of baseline risk factors

All of the outpatient records were screened for patients who required treatment for DM (ICD-9 code 250) and hypertension (ICD code 401-405) on two or more occasions.

### Survival status

The survival status and cause of death in the event of fatality of the subjects between 2000 and 2008 were ascertained by using the National Death Registry in Taiwan, which records the cause of death for all deceased citizens.

### Statistical analysis

Subjects were followed up from 1 January 2000 to the date of issuance of ESRD certificates or death or the end of the study (31 December 2008), whichever was earliest. The ESRD incidence was calculated as the ratio of the number of incident ESRD cases to the population count in LHID 2000 and was expressed as the annual incidence per population of 1,000.

The difference in the incidence of ESRD among patients with gout and those without gout was analyzed by using the log-rank test. Cox proportional hazards models were constructed with the dichotomous variable denoting whether the patient had ESRD during the study period. Covariates used in the models included age, sex, and history of DM or hypertension between 1996 and 1999. Hazard ratios (HRs) and 95% confidence intervals (CIs) were computed. A two-sided *P *value of less than 0.05 was considered statistically significant. All analyses were performed by using PASW Statistics, version 18, and PASW Modeler, version 13 (SPSS Inc., Chicago, IL, USA).

## Results

After exclusion of subjects with a history of ESRD, CAD, or stroke, we selected 656,108 subjects (336,733 men and 319,375 women) who were at least 20 years old. Gout was diagnosed in 19,963 (3.0%) of the patients between 1996 and 1999; 14,542 (72.8%) were male. The mean age of the patients was 41.1 ± 15.6 years, and there was no difference between the sexes. However, patients with gout were significantly older than the others (52.2 ± 15.2 versus 40.7 ± 15.4 years).

Table 1 summarizes and compares the baseline characteristics between patients with gout and those without gout. In general, patients with gout were significantly more likely to develop DM and hypertension, and odds ratios (ORs) were 5.94 and 7.21, respectively.

**Table 1:** Baseline characteristics of patients and risk of diabetes mellitus or hypertension or both by gout status

	Gout (*n *= 19,963)	Non-gout (*n *= 636,145)	Odds ratio (95% CI)
Age in years, mean ± SD	52.2 ± 15.72	40.7 ± 15.4	
Males, number (percentage)	14,542 (72.8%)	322,191 (50.6%)	
Years of follow-up, mean ± SD	8.0 ± 0.4	8.0 ± 0.2	
DM, number (percentage)	2,619 (13.1%)	18,507 (2.9%)	5.94 (4.82-5.26)
Hypertension, number (percentage)	6,311 (31.6%)	38,336 (6.0%)	7.21 (7.00-7.44)
DM or hypertension, number (percentage)	7,367 (37.4%)	49,190 (7.7%)	7.13 (6.92-7.35)
DM and hypertension, number (percentage)	1,463 (7.3%)	7,653 (1.2%)	6.49 (6.13-6.88)

CI, confidence interval; DM, diabetes mellitus; SD, standard deviation.

The cohort was followed for a total of 5,242,530 person-years from January 2000 for a mean follow-up period of 8.0 ± 0.2 years. During the study period, ESRD was diagnosed in 2,377 patients (276 with gout and 2,101 without gout) and chronic renal replacement therapy was required. The incidences of ESRD among patients with gout and those without gout were 1.73 and 0.41 per 1,000 person-years (log-rank test, *P *< 0.001), respectively. The incidence of ESRD did not differ between the sexes.

Next, we conducted multivariate Cox proportional models to determine the adjusted HR for ESRD in gout. Gout was associated with an age- and sex-adjusted HR (95% CI) of 1.75 (1.59 to 1.94) for ESRD. After adjustment for age, sex, and history of DM and **} **hypertension, gout was associated with an HR of 1.57 (95% CI 1.38 to 1.79; *P *< 0.001) for ESRD (Table 2).

**Table 2:** The association between gout and the occurrence of end-stage renal disease

Covariates	HR (95% CI)	*P *value
Gout	1.57 (1.38-1.79)	< 0.001
Age	1.027 (1.02-1.03)	< 0.001
Male gender	1.04 (0.96-1.12)	0.395
Diabetes mellitus	6.20 (5.61-6.85)	< 0.001
Hypertension	2.18 (1.97-2.42)	< 0.001

CI, confidence interval; HR, hazard ratio.

In patients with ESRD, 861 individuals - 77 with gout (27.9%) and 521 without gout (24.8%) - died due to ESRD. The presence of gout did not increase the risk of death to a level greater than that in those without gout. The log-rank test showed no survival difference between ESRD patients with gout and those without gout (*P *= 0.21). After adjustment for age, sex, and history of DM and hypertension, gout was associated with an adjusted HR of 0.95 (95% CI 0.74 to 1.26; *P *= 0.71) for mortality.

Among patients without DM and hypertension (*n *= 599,450), a higher risk of ESRD was also observed in patients with gout than in those without gout. During 4,792,542 person-years of follow-up, 1,132 subjects developed ESRD (70 with gout and 1,062 without gout), corresponding to rates of incidence of ESRD of 0.70 and 0.22 per 1,000 person-years (log-rank test; *P *< 0.001) for patients with gout and those without gout, respectively. Among subjects without DM and hypertension, gout was associated with an age- and sex-adjusted HR of 2.00 (95% CI 1.56 to 2.56; *P *< 0.001) for ESRD.

Next, we explored the association between gout and ESRD in three age groups (20 to 44 years, 45 to 59 years, and at least 60 years). Risk for ESRD in patients with gout was higher among older subjects. For those who were 20 to 44 years old, gout was not independently associated with ESRD after adjustment for age, sex, DM, and hypertension. However, gout was associated with HRs of 1.41 and 1.58 for ESRD in patients who were 45 to 59 years old and those at least 60 years old, respectively (Table 3).

**Table 3:** Risk for end-stage renal disease among patients with gout, stratified by age group

	20-44 years (*n *= 419,298)	45-59 years (*n *= 115,171)	60 years or older (*n *= 64,981)
Gout, number (percentage)	5,832 (1.4%)	3,989 (3.5%)	2,675 (4.1%)
ESRD, gout/non-gout	16/397	26/364	28/301
HR (95% CI) for ESRD	1.27 (0.91-1.78)	1.41 (1.13-1.76)	1.58 (1.32-1.90)

CI, confidence interval; ESRD, end-stage renal disease; HR, hazard ratio.

## Discussion

Investigation of data recorded for a representative cohort of the general population in Taiwan revealed that gout was independently associated with an HR of 1.57 for ESRD after adjustment for age, sex, and history of DM and hypertension. Among subjects without a history of DM or hypertension, the risk for ESRD in patients with gout was twofold that in those without gout. The risk of ESRD was not significantly high for younger patients (20 to 44 years old) but increased with age after 45 years. Mortality among patients with ESRD was not affected by the presence or absence of gout. Since patients with gout were 57% more likely to develop ESRD, the specific risk for death due to ESRD remained higher in the gout-affected population than in the normal population. This result was consistent with our recent finding that gout was associated with a threefold standardized mortality ratio for kidney disease [19]. Overall, gout is associated with a greater hazard of development of ESRD.

CKD is an important public health problem. The direct and indirect costs of caring for patients with CKD are enormous, particularly for those requiring chronic renal replacement therapy [20]. Fortunately, early detection and prompt treatment of CKD may prevent or delay its complications [21]. However, because it is frequently asymptomatic, early-stage CKD is often overlooked [22]. Proteinuria [23], hypertension [24], hyperglycemia [25], and metabolic syndrome [26] are well-documented risk factors for CKD and are fairly common in patients with gout. Consistent with these findings, this study showed that gout was associated with a sixfold risk for DM and a sevenfold risk for hypertension. Our previous study also revealed higher percentages of proteinuria in patients with gout (9.8%) than in subjects with hyperuricemia (7.8%) or normouricemia (4.4%) [27]. Since the clinical presentation of gout is more prominent than that of the other risk factors of CKD, a diagnosis of gout should be regarded as a warning sign for CKD and a thorough assessment of kidney function is warranted.

Among patients with ESRD, the mortality of patients with gout was similar to that of patients without gout. This finding seems to be contrary to that of a previous study, which reported that gout was associated with an adjusted HR of 1.49 for mortality [28]. However, the case definition for gout in that study was different: we evaluated gout patients before they developed ESRD, whereas Cohen and colleagues [28] evaluated patients both developing gout and undergoing dialysis treatment. Gout incidence was thought to be low among patients with ESRD [29], probably because inflammatory mediators are removed by dialysis [30]. Incident gout after initiation of dialysis may represent a higher metabolic disturbance or level of inflammation among patients with ESRD and therefore leads to an increased risk of death. Further investigation is required to confirm this.

The reason for the increased risk of ESRD among people with gout is unclear. Hyperuricemia has been linked to reduced renal function and the development and progression of renal disease. In a previous study, hyperuricemia was found to be associated with an adjusted OR of 1.82 for the deterioration of kidney function [31]. Another study indicated that, for every 2-mg/dL increase in serum uric acid level, the OR for new-onset kidney disease was 1.69 [13]. A uric acid level of 7.0 to 8.9 mg/dL was reported to be associated with an OR of 1.74 for incident kidney disease after adjustment for baseline glomerular filtration rate [32]. A recent trial randomly assigned 54 patients with CKD to allopurinol treatment or control groups and demonstrated that a reduction in serum uric acid levels mitigated the progression of CKD [33]. Besides coexisting with hyperuricemia, gout often coexists with hypertension and DM, as was the case in the present study. Moreover, patients with gout are more likely to have received non-steroid anti-inflammatory drugs, which have been documented to be a risk factor for ESRD [34].

Use of administrative databases enables population-based epidemiologic studies; however, limitations exist. The inability to apply clinical case definitions, particularly in a common complex disease such as gout, is one of the inherent limitations. Currently, there are three classification criteria for gout [35-37]. In many cases (in particular, in studies based on administration databases), none of the aforementioned criteria can be applied, because of the lack of clinical detail and laboratory parameters, regardless of how good the differential power of the previous classification criteria may be. In such settings, the identification of gout cases often depends on patient recall or physician diagnosis. For self-report physician diagnosis of gout, a recent study suggests that it has good reliability and sensitivity [38]. The utility and validity of gout diagnoses in claims data had also been assessed [17,18]. Harrold and colleagues [17] demonstrated that gout diagnosis on two or more occasions portended a positive predictive value of 61%. In another study, Jasvinder and colleagues [18] found that ICD-9 codes for gout had a sensitivity of 90%, a specificity of 100%, and positive and negative predictive values of 100% and 87%, respectively. Recently, Mikuls and colleagues [39] used a case definition based on the use of anti-gout treatment. Using both diagnosis and treatment to define a patient with gout should result in a case definition that is more stringent than one that defines cases on the basis of diagnoses only. The decision of case definition in epidemiological studies should be made on an individual basis. In this study, two case definitions were used, and they produced similar results. This reinforces our conclusion that patients with gout have a higher risk of ESRD.

Other limitations of this study merit discussion. First, we had no information on some potential covariates, such as serum uric acid or creatinine levels. Socioeconomic status has been mentioned as a risk factor for ESRD [40]; however, there was no proper surrogate marker to represent socioeconomic status in the database. Therefore, these factors could not be included in the modeling process. Second, the use of administrative claims data also precluded adjustment for lifestyle covariates, such as diet and smoking. Despite these limitations, the results can be generalized and are applicable to the general population because the study is population-based. Furthermore, since the primary data source was claims data of the Taiwan NHIRD, which covers almost the entire population in Taiwan, and the information regarding exposure, outcome, and covariates was prospectively collected, the possibility of missing data or loss to follow-up is almost non-existent.

## Conclusions

The present study demonstrated that gout independently increased the hazard of development of ESRD, and the magnitude of hazard in the gout-affected population was 57% higher than that in the general population. The risk was significant even among patients who had gout but not DM or hypertension. These findings justify the careful monitoring of renal function in patients with gout.

## Abbreviations

CAD: coronary heart disease; CI: confidence interval; CKD: chronic kidney disease; DM: diabetes mellitus; ESRD: end-stage renal disease; HR: hazard ratio; ICD-9: International Classification of Diseases: Ninth Revision; LHID: Longitudinal Health Insurance Database; NHI: National Health Insurance; NHIRD: National Health Insurance Research Database; OR: odds ratio; NHRI: National Health Research Institute.

## Competing interests

The authors declare that they have no competing interests.

## Authors' contributions

C-FK participated in the design of the study, helped to perform the statistical analysis, helped to conceive of the study and participated in its design and coordination, and helped to write the draft of the manuscript. L-CS, K-HY, and H-CC participated in the design of the study. M-JC helped to perform the statistical analysis. I-JC helped to conceive of the study, participated in its design and coordination, and helped to write the draft of the manuscript. All authors participated in the production of the final manuscript. All authors read and approved the final manuscript.
